# Evaluation of the Performance of the Novodiag^®^ Stool Parasites Assay for the Detection of Intestinal Protozoa and Microsporidia

**DOI:** 10.3390/pathogens12070889

**Published:** 2023-06-29

**Authors:** Pamela Chauvin, Florie Barba, Emilie Guemas, Eléna Charpentier, Claire Cottrel, Judith Fillaux, Alexis Valentin, Sarah Baklouti, Sophie Cassaing, Sandie Ménard, Antoine Berry, Xavier Iriart

**Affiliations:** 1 Service de Parasitologie—Mycologie, Centre Hospitalier Universitaire de Toulouse, 31059 Toulouse, France; 2 Institut Toulousain des Maladies Infectieuses et Inflammatoires (Infinity), Université de Toulouse, CNRS UMR5051, INSERM UMR1291, Université Paul Sabatier, 31024 Toulouse, France; 3 RESTORE Institute, UMR 1301-Inserm 5070-CNRS EFS Université Paul Sabatier, 31100 Toulouse, France; 4 UMR 152 PHARMA-DEV, IRD, UPS, Université de Toulouse, 31062 Toulouse, France; 5 Laboratoire de Pharmacocinétique et Toxicologie, CHU de Toulouse, 31059 Toulouse, France; 6 INTHERES, Université de Toulouse, INRAE, ENVT, 31076 Toulouse, France

**Keywords:** stool, protozoa, microsporidia, diagnosis, microscopy, qPCR, Novodiag Stool Parasites, performance

## Abstract

Objectives: We aimed to assess the performance of the Novodiag^®^ Stool Parasites (NSP) assay in the diagnosis of the most common intestinal protozoan and microsporidia infections. Methods: A panel of 167 selected stool samples was retrospectively analysed with the NSP assay and compared to routine microscopy and qPCR methods for the detection of pathogenic protozoa and microsporidia. Results: Whereas specificity was high for all protozoa and microsporidia, NSP sensitivity was strongly dependent on the comparative method used as reference. When compared to microscopic methods, NSP sensitivity was high (96.7 to 100%) for *Blastocystis hominis*, *Entamoeba histolytica* and *Cyclospora cayetanensis* but was lower for *Giardia intestinalis* (85.2%) and ≤50% for *Cystoisospora belli* and *Dientamoeba fragilis*. In comparison to conventional qPCR, the NSP assay demonstrated lower sensitivity characteristics dependent on parasite loads, reaching 60 to 70% for *G. intestinalis*, *D. fragilis*, *Cryptosporidium* spp. and *E. histolytica.* Sensitivity was 100% for *Enterocytozoon bieneusi*, but none of the five samples containing *Encephalitozoon* spp. were detected. Conclusions: The overall performance of the NSP assay in the diagnosis of gastrointestinal protozoa and microsporidia seems to be better than or equivalent to that observed with microscopic methods but inferior to that obtainable with classical targeted qPCR.

## 1. Introduction

For many years, microscopic examination has been the only tool available for the detection of gastrointestinal parasites in stool specimens and remains the cornerstone for the diagnosis of parasitic infections in most routine diagnostic laboratories. However, this approach has some limitations because it is labour-intensive and requires a high level of skill for optimal examination, which remains a major challenge due to the low number of positive samples received annually for many laboratories in high-income countries. Microscopic methods may produce poor analytical sensitivity, and it is therefore usually accepted that three consecutive samples collected over a few days are necessary to increase its sensitivity [1]. Finally, microscopic examination is also ineffective in differentiating pathogenic and non-pathogenic members of some species complex of protozoans such as *Entamoeba*, and it is unable to diagnose microsporidia or coccidia (Cryptosporidia, *Cyclospora cayetanensis*, *Cystoisospora belli*) without specific staining.

As a consequence, there is growing interest in alternative methods such as DNA detection mostly by real-time PCR to overcome the limitations of microscopy. Compared with microscopy, DNA-based detection methods display several advantages such as an increased sensitivity and specificity, the opportunity for syndromic approach development, the possibility for molecular typing and an optimised turnaround time when PCR is coupled with automated DNA extraction [2,3]. Nevertheless, one of the current challenges is to develop DNA-based detection methods which can cover the diversity of gastrointestinal parasites infecting the digestive tract.

The Novodiag^®^ Stool Parasites (NSP) assay is a new automated approach combining real-time PCR and microarray technologies. This diagnostic test detects the presence of nucleic acids in 26 targets from stool samples including the most common protozoans, helminths and microsporidia (Supplementary Data: Table S1).

In European countries, epidemiological studies reported around 19% of intestinal parasitic infections [4]. Intestinal infections with helminths had a low prevalence (around 1.4%) [4] compared to infections due to protozoa and microsporidia (around 17%) [4,5]. *Blastocystis* sp. was the most frequently detected species (10.5 to 13.9%), which was followed by *Dientamoeba fragilis* (1.7 to 2.3%) and *Giardia intestinalis* (1.89 to 2.3%) [4,5]. In Europe, the prevalence of other parasites was generally <1% each [4]. Thus, pathogenic protozoan and microsporidia species account for about 95% of gastrointestinal parasites diagnosed every year in hospital microbiology laboratories in Europe [6]. New marketed tests must therefore perform well on these targets, which are very frequently encountered in the routine practice of medical laboratories.

To date, there has been only one published study evaluating the performance of the NSP assay in the diagnosis of intestinal parasitosis performed in comparison to microscopy [7]. Here, our aim was to evaluate the performance of the NSP multiplex PCR assay for the detection of the most common intestinal pathogenic protozoa and microsporidia in comparison with routine microscopy methods and conventional molecular assays (Amplidiag^®^ Stool Parasites real-time PCR kit and in-house qPCR for microsporidia).

## 2. Materials and Methods

### 2.1. Sample Collection

The study was carried out on a panel of 167 stool samples submitted to the parasitology-mycology unit of the Toulouse University Hospital (France) between February 2018 and June 2022. Of the 167 stool samples, 135 were analysed by routine molecular analysis (real-time PCR) and NSP assay at the time of patient management. Given the rarity of some targets, 32 frozen (−20 °C) samples were added to the panel. For these 32 samples, real-time PCR and NSP assay were performed simultaneously after thawing the archived stool samples. Stool samples were selected to obtain the widest possible variety of protozoan or microsporidia species targeted by the NSP assay and the most variable microorganism loads possible.

All these stool samples came from patients who had undergone a parasitological stool examination and/or a targeted search for cryptosporidia and/or microsporidia. Samples were obtained only for routine diagnosis on the basis of doctors’ prescriptions. According to French Public Health Law [8], this protocol did not require Ethics Committee approval and was exempt from the requirements for formal informed consent.

The evaluation of NSP performance was only assessed on the detection of protozoa and microsporidia considered potentially pathogenic for humans (*Blastocystis* sp., *Cystoisospora belli*, *Dientamoeba fragilis*, *Entamoeba histolytica*, *Giardia intestinalis*, *Cryptosporidium* spp., *Cyclospora cayetanensis*, *Balantioides coli*, *Encephalitozoon* spp. and *Enterocytozoon bieneusi)*. Stools containing only helminths or non-pathogenic protozoa not targeted by the NSP assay (i.e., non-pathogenic amoeba species and flagellates) were considered negative samples.

### 2.2. Routine Microscopy for Intestinal Parasites

Routine microscopic examination of each stool sample was performed upon receipt of the sample by experienced microscopists after staining with merthiolate–iodine–formaldehyde colouration [9] and concentration using the Ovatec^®^ Plus flotation technique (Zoetis) and Bailenger method (Para-selles^®^ optima—Bailenger, Biosynex^®^, Illkirch-graffenstaden, France) according to the manufacturer’s recommendations.

When a protozoan of the *Entamoeba histolytica*/*dispar* complex was identified by routine microscopy, a precise determination of the involved species was performed by the Amplidiag^®^ Stool Parasites assay (Hologic formerly Mobidiag, Espoo, Finland). The detection of *Cryptosporidium* spp. and microsporidia relied only on specific PCR assays (see below). The presence of *Cystoisospora belli* in a stool sample was first suspected after routine microscopic examination and confirmed using a specific PCR [10]. The diagnosis of *Cyclospora cayetanensis* infections was performed using a fluorescence microscope (UV at 365 nm) (autofluorescence) and confirmed using a specific PCR [11].

### 2.3. Routine Stool DNA Extraction and Multiplex Real-Time PCR

For each patient, a 250 to 500 mg of stool sample (or 250 µL for liquid stool) was suspended in 1 mL of phosphate-buffered saline (PBS) and homogenised by bead beating at 7000 rpm for 90 s (MagNA Lyser, Roche Diagnostics, Mannheim, Germany). DNA extraction was performed using the High Pure PCR Template Preparation Kit (Roche Diagnostics, Meylan, France) according to the manufacturer’s instructions. Briefly, 200 µL of binding buffer and 50 μL proteinase K were added to 200 μL of stool suspension. After a ten-minute incubation at 70 °C, 100 μL of iso-propanol was added, and the solution was centrifuged through a filter tube for 1 min at 8000× *g*. The filter tube was subsequently centrifuged for 1 min at 8000× *g* after adding 500 μL of inhibitor removal buffer and washed three times with wash buffer. The DNA was then eluted in 200 μL of elution buffer by centrifuging for 1 min at 8000× *g*.

Routine molecular detection of *Giardia intestinalis*, *Dientamoeba fragilis*, *Cryptosporidium* spp. and *Entamoeba histolytica* was performed using the Amplidiag^®^ Stool Parasites real-time PCR kit (Hologic formerly Mobidiag, Espoo, Finland) according to the manufacturer’s recommendations. The Amplidiag^®^ Stool Parasites real-time PCR kit comes with a calibration kit for Amplidiag^®^ Stool Parasites (AD-SPC-30), and each run includes positive and negative controls. After PCR amplification on a CFX96 instrument (Bio-Rad, Richmond, VA, USA), data were analysed with the Amplidiag^®^ Analyser software (Hologic formerly Mobidiag, Espoo, Finland) using internal thresholds. For the study, in order to avoid potential false-positive results, all samples with a Ct ≥ 36 (regardless of target) were run at least in duplicate. In case of positive PCR for *Cryptosporidium* spp., species determination was performed by molecular sequencing on an ABI 3130 XL (Applied Biosystem, Waltham, MA, USA) using the primers described by Mary et al. [12]. Routine molecular detection of microsporidia was performed on a LightCycler 480 instrument (Roche Diagnostic, Mannheim, Germany) according to the protocol described by Polley et al. [13] which allows the detection of *Enterocytozoon bieneusi and Encephalitozoon* spp.

### 2.4. Novodiag^®^ Stool Parasites Assay

Sample preparation and Novodiag^®^ Stool Parasites assay (Hologic formerly Mobidiag, Espoo, Finland) was performed according to the manufacturer’s instructions. In brief, the stool was collected with a sterile swab (eSwab tube, Copan, Brescia, Italy) from several places in the faeces. The swab was then placed in the eSwab tube and vortexed for 5 s. In case of liquid stool, 200 μL was taken and then directly added into the eSwab tube.

The entire contents of the eSwab tube were then transferred into a MagNALyser tube (Roche Diagnostics, Mannheim, Germany). After bead beating at 7000 rpm for 90 s (MagNALyser, Roche Diagnostics, Mannheim, Germany), 600 μL of the lysate was then transferred into the NSP cartridge using a sterile single-use pipette under a class II biological safety cabinet (laminar flow). The cartridge was inserted into the Novodiag instrument, and the results (Positive/Negative/Invalid) were automatically displayed on the instrument screen at the end of the reaction.

According to the manufacturer’s instructions, every sample with an invalid result was retested a second time with a new test tube (new eSwab tube from the original sample) and, if needed, a third time with a diluted sample (300 μL of the first eSwab tube transferred into a second eSwab tube).

### 2.5. Statistical Analysis

Data were analysed using SIGMA Stat1 software (2.03; Jandel Corporation, San Jose, CA, USA). Data were expressed as mean ± standard deviation. A Student’s t test was used for comparisons between two groups. For proportion comparisons, a chi-square test was used. A kappa coefficient was used to measure the agreement between each test. Differences were considered statistically significant if the *p*-value was < 0.05.

## 3. Results

### 3.1. Valid Sample for Analysis

**Figure 1:**
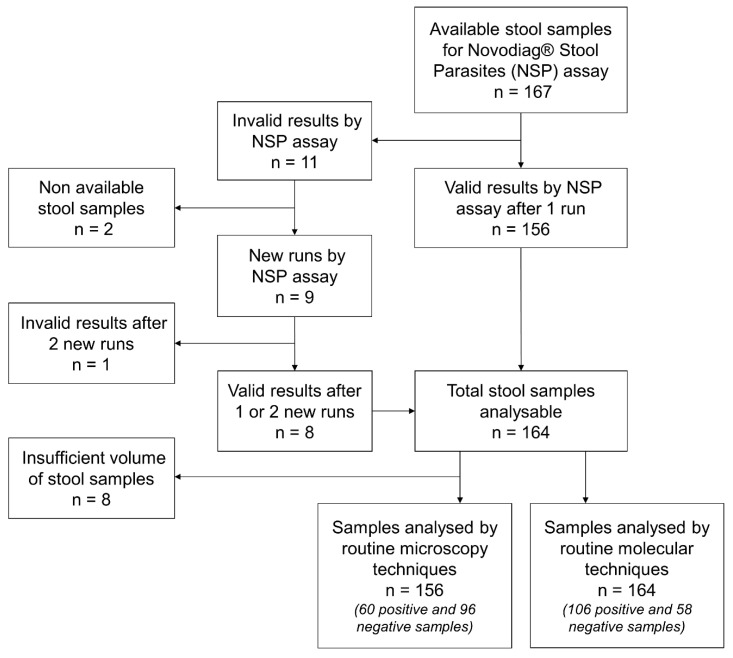
Flow Chart of Samples.

One hundred and sixty-seven stool samples were analysed by the NSP assay for the detection of intestinal protozoa or microsporidia (Figure 1). Eleven stool samples (6.6%) showed invalid results (no amplification of an internal control DNA) by NSP assay. The invalid results seemed to be independent from the consistency of the baseline stool and the storage temperatures of the samples (fresh or frozen stools). Of the nine samples that could be retested, seven were valid after a second run and one was valid after a third test (diluted condition). For one sample, the result remained invalid after three tests. Finally, 164 stool samples could be analysed and compared with routine microscopy (*n* = 156) or molecular techniques (*n* = 164).

### 3.2. Performance of the Novodiag^®^ Stool Parasites Assay Compared to Routine Microscopy Techniques

Of the 156 stool samples analysed by both NSP assay and routine microscopy techniques, 60 were microscopically positive for at least one potential pathogenic protozoan and 96 stools samples were considered negative (74 without any protozoa, 6 only with helminths and 16 only with non-pathogenic amoeba or flagellate species). As expected, the 16 microscopically positive stools with only non-pathogenic protozoa (*Entamoeba dispar*, *Entamoeba coli*, *Endolimax nana* and *Chilomastix mesnili*), which were not among the targets detected by NSP, were found to be negative by this assay.

The specificity (Sp) of the NSP kit compared to routine microscopy techniques was excellent for *Cyclospora cayetanensis* (Sp: 100%), *Cystoisospora belli* (Sp: 100%), *Giardia intestinalis* (Sp: 97.7%) and *Entamoeba histolytica* (Sp: 96.1%) (Table 1). For *Blastocystis hominis*, specificity was 77.8% as 28 additional *Blastocystis hominis* samples were positive by the NSP assay. The same was true for *Dientamoeba fragilis* where 22 additional samples were detected positive by the NSP method.

The sensitivity (Se) of the NSP kit compared to microscopy techniques was excellent for *Entamoeba histolytica* (Se: 100%), *Cyclospora cayetanensis* (Se: 100%), and *Blastocystis hominis* (Se: 96.7%) (Table 1). The sensitivity of the NSP kit for *Giardia intestinalis* was 85.2%. For the four samples not detected by the NSP assay, positivity for *Giardia intestinalis* was confirmed by routine Amplidiag^®^ PCR assay (Ct: 25.4–33.0). These four stools corresponded to polyparasitic samples with two or three protozoa detected by routine microscopy and/or Amplidiag^®^ PCR methods. In these stools, the NSP method correctly detected the other protozoa associated with the missed *Giardia intestinalis*. For *Dientamoeba fragilis*, only one of two microscopically positive samples was detected. Positivity for *Dientamoeba fragilis* in the sample missed by the NSP assay was confirmed by routine Amplidiag^®^ PCR assay (Ct: 32.5). Only one positive sample for the *Cystoisospora belli* was tested in duplicate but was not detected by the NSP assay. No samples with *Balantioides coli* were available in the laboratory during the inclusion period.

In order to assess the influence of the storage temperature on the NSP performance, the proportion of intestinal protozoa not detected in fresh and frozen stool samples was compared. No significant difference was observed between fresh (5.1%; 6/124) and frozen (3.1%; 1/32) stool samples (*p* = 0.676) with microscopy techniques as reference.

### 3.3. Performance of the Novodiag^®^ Stool Parasites Assay Compared to Routine Molecular Biology Techniques

Of the 164 stool samples analysed by both NSP assay and routine molecular biology techniques, 106 were positives by routine qPCR for at least one pathogenic protozoan or microsporidia, and 58 stools samples were negative.

**Figure 2:**
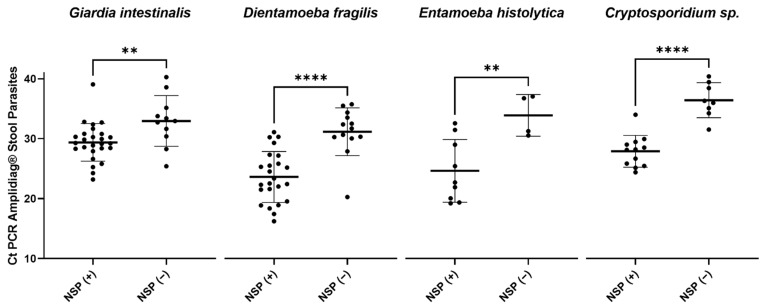
Ct values from Amplidiag^®^ Stool Parasites PCR for *Giardia intestinalis*, *Dientamoeba fragilis*, *Entamoeba histolytica* and *Cryptosporidium* spp., according to the detection status from the Novodiag^®^ Stool Parasites (NSP) Assay. An individual and specific PCR for each species *Giardia intestinalis*, *Dientamoeba fragilis*, *Entamoeba histolytica*, and *Cryptosporidum* spp. was performed with the Amplidiag^®^ Stool Parasites kit. For each of the 4 targets, Ct values have been ranked according to the NSP results. NSP(+): samples detected by the NSP assay. NSP(−): samples not detected by the NSP assay. ** *t*-test *p* < 0.01; **** *t*-test *p* < 0.0001.

The specificity of the NSP assay compared to routine molecular biology techniques was excellent (Sp: 100%) for the four protozoa and two microsporidia species tested (Table 2). Sensitivities ranged from 60.0% to 70.3% depending on the protozoan (Table 2). For *Giardia intestinalis*, *Entamoeba histolytica*, *Cryptosporidium* spp., and *Dientamoeba fragilis*, the false negative samples produced by the NSP kit corresponded to the lowest parasite loads according to Ct measured by PCR Amplidiag^®^ Stool parasites assay (*Giardia intestinalis n*= 11/37, Ct: 25.4–40.3; *Entamoeba histolytica n*= 4/13, Ct: 30.5–37.1; *Cryptosporidium* spp. *n*= 8/20, Ct: 31.5–40.4 and *Dientamoeba fragilis n*= 14/38, Ct: 20.3–36.0 *p* < 0.05; Figure 2).

For *Giardia intestinalis*, the 26 positive stools correctly detected by the NSP assay (Se: 70.3%) had low Ct values (i.e., high parasite load) in Amplidiag^®^ Stool Parasites PCR (Ct: 23.2–39.1) and were all positive by routine microscopy. In addition, among the 11 stools not detected by the NSP assay, 4 were positive by microscopy.

The sensitivity of the NSP assay in detecting *Dientamoeba fragilis* was 63.1% among the 38 samples detected positive by routine Amplidiag^®^ PCR assay, but about 95% (36/38) of these samples produced negative results by microscopy.

Of the 13 stools positive for *Entamoeba histolytica* with Amplidiag^®^ Stool Parasites PCR (Ct: 19.2–37.1), 9 stools were correctly detected by the NSP assay (Se: 69.2%) corresponding to the samples with low Ct values (i.e., high parasite loads) by Amplidiag^®^ Stool Parasites PCR (Ct: 19.2–32.6). Among the nine samples detected, only three were positive for *Entamoeba histolytica* cysts by microscopic examination. The four stool samples not detected by the NSP assay had high Ct values (i.e., low parasite loads) for *E. histolytica* (Ct: 30.5–37.1) and were all negative by routine microscopy.

Concerning *Cryptosporidium* spp., among the 20 positive stools detected by Amplidiag^®^ Stool Parasites PCR (Ct: 24.4–40.4), 12 were positive by the NSP assay (Se: 60.0%) and also corresponded to low Ct values (i.e., high parasite loads) (Ct: 24.4–34.0, Figure 2). Eighteen samples could be sequenced for *Cryptosporidium* species identification, showing that detection was possible for at least one sample of *Cryptosporidium parvum*, *C. hominis* and *C. felis*. As expected, the *Cryptosporidium canis* positive stool (Ct: 31.5) was not detected by the NSP assay, as this species does not belong to the targets detected by the kit (Supplementary Data: Table S1). Only two samples with *Cryptosporidium parvum* were not detected by the NSP method corresponding to the lowest Ct values (Ct: 35.1 and 39.5). For *Cryptosporidium hominis* and *felis* species, the samples not detected by the NSP assay had high Ct values (i.e., low parasite loads) (*C. hominis*: Ct: 34.2 and 38.4; *C. felis*: Ct: 36.0).

Finally, all the nine stool samples positive for *Enterocytozoon bieneusi* by in-house qPCR (Ct: 17.6–34.5) were detected by the NSP assay (Se: 100%). For *Encephalitozoon* spp., none of the five positive stool samples (Ct: 22.9–40.0) were detected by the NSP kit, even after retesting. Of the five stools, two had high Ct values (Ct: 36.1–40) with routine PCR, indicating a probable low parasite load in these samples. The three other undetected stools had Ct values between 22.8 and 25.7 (i.e., high parasite loads) but also contained *Giardia intestinalis* ± *Blastocystis hominis* in addition to microsporidia. In these three stools, the NSP assay correctly detected *Giardia intestinalis* and *Blastocystis hominis* associated with the missed *Encephalitozoon* spp.

In order to assess the influence of the storage temperature on the NSP performance, the proportion of intestinal protozoa not detected in fresh and frozen stool samples was compared. No significant difference was observed between fresh (25%; 33/132) and frozen (21.9%; 7/32) stool samples (*p* = 0.712) with routine molecular biology techniques as reference.

### 3.4. Comparison of the Sensitivity of the Novodiag^®^ Stool Parasites Assay and Routine Microscopy Techniques with Routine Molecular Biology Technique as Reference

To evaluate the performance of the NSP assay and routine microscopy, the sensitivity of detection for *Giardia intestinalis*, *Dientamoeba fragilis* and *Entamoeba histolytica* was compared using the routine molecular biology technique as reference. Of the 156 samples that were tested by both microscopy and routine PCR, the sensitivity of the NSP kit was significantly higher than that of microscopy in the detection of *Dientamoeba fragilis* and *Entamoeba histolytica* (*p* < 0.001 and *p* = 0.018, respectively) (Table 3). Sensitivity was not statistically different for *Giardia intestinalis* between the two techniques (*p* = 0.797) (Table 3).

## 4. Discussion

The molecular diagnosis of intestinal parasitic infections has long been limited to a few parasitic targets due to the large number and wide variety of parasites needing detection. Today, several tests allow the simultaneous detection of a large number of intestinal parasites including helminths [14,15,16]. Among these kits, the NSP assay, which is based on a detection system combining real-time PCR and DNA chip technologies, enables the detection of 26 distinct targets.

While molecular detection kits for protozoa are increasingly implemented in laboratories, the only published study reporting the performance of the NSP assay in the diagnosis of intestinal parasitosis was performed in comparison with microscopy [7]. One of the strengths of our work lies in the fact that the performance of the NSP assay for the detection of the most common intestinal pathogenic protozoa and microsporidia was also compared with other molecular methods.

In accordance with the study by Hartuis et al. [7], the specificity of the NSP assay was very high (100%) for *Giardia intestinalis*, *Dientamoeba fragilis*, *Entamoeba histolytica*, *Cryptosporidium* spp., microsporidia, *Cyclospora cayetanensis* and *C. belli* when compared to routine molecular methods or microscopy (when PCR were not available for the target). Only the target species *Blastocystis hominis* had 77.8% specificity compared to microscopy alone. However, as the sensitivity of microscopy is low and the morphological diagnosis of this species is difficult, it can be assumed that microscopy largely underestimates the detection of this species. The microscopic examination of trichrome stained stool samples could have improved the detection of *Dientamoeba fragilis* and *Blastocystis* sp. However, this work was based on routine techniques (MIF and Bailenger) much more commonly used in medical laboratories than trichrome staining for the parasitological examination of stools. Nevertheless, for both *Blastocystis hominis* and *D. fragilis*, the relevance of detecting these species is relative because the pathogenicity of these two protozoa is currently discussed [17]. Finally, 16 stools that were microscopically positive for *Entamoeba dispar*, *Entamoeba coli*, *Endolimax nana* and *Chilomastix mesnili*, were not detected with the NSP assay. This suggests an absence of cross-reactivity with these non-pathogenic protozoa not targeted by the NSP assay but commonly found in stools.

NSP sensitivity was highly dependent on the comparative method used as reference. When compared to the microscopic method, sensitivity was high, reaching 96.7 to 100% for *Blastocystis hominis*, *Entamoeba histolytica* and *Cyclospora cayetanensis.* Sensitivity was ≤50% for *Cystoisospora belli* and *Dientamoeba fragilis*, but the number of microscopically positive samples was low in our study for these targets. Although sensitivity for *Giardia intestinalis* was rather good compared to microscopy (Se: 85.2%), the effectiveness of the NSP assay may seem disappointing compared to the usual performance of other molecular biology techniques for this target [5,14,18,19]. This impairment in the detection of *Giardia intestinalis* observed in our study could be explained by the concomitant presence of several NSP targets in the same sample and by a possible competition phenomenon in molecular amplification and detection between these targets. This lack of sensitivity for *Giardia intestinalis* detection was already reported by Hartuis et al. [7].

By comparison to conventional molecular biology techniques, the NSP assay performed less well for protozoan detection, reaching 60 to 70% for *G. intestinalis*, *D. fragilis*, *Cryptosporidium* spp. and *E. histolytica.* For these four species, NSP detection probably depended on parasite loads as the NSP assay did not detect the highest Ct values (low parasite load) found by routine qPCR. Except for *Cryptosporidium canis* which is not targeted by the NSP assay, the species *Cryptosporidium parvum*, *C. felis* and *C. hominis* were detected at least once. For *Cryptosporidium*, non-detection of the target seems to depend mainly on the parasite load and not on the species in question (except for *Cryptosporidium canis*, known to be untargeted). *Enterocytozoon bieneusi*, the most frequently observed microsporidia species, was detected with 100% sensitivity. Nevertheless, the genus *Encephalitozoon* spp. was not found in the five different samples evaluated despite a high load of microorganisms in three of them. As for *Giardia intestinalis*, a detection failure of this target due to the presence of many parasites in the samples cannot be excluded (possible competition between the targets). Although less common than *Enterocytozoon bieneusi*, this lack of sensitivity for *Encephalitozoon* spp. could be a problem in immunocompromised patient populations.

Our results demonstrated that although the NSP assay is a DNA-based system, it does not achieve the sensitivity of targeted qPCR in the detection of protozoa and microsporidia. Nevertheless, for the parasite targets assessed by both routine PCR and microscopy methods, our study showed that the NSP assay was much more sensitive than microscopy for *Dientamoeba fragilis* and *Entamoeba histolytica* (21 and 6 additional samples detected, respectively). For *Giardia intestinalis*, sensitivity was not statistically different between NSP and microscopy.

The NSP technique covers a large majority of the parasites present in the stool and allows complete results to be obtained within 90 min with a *random-*access** fully automated system. The technical handling time without prior DNA extraction is reduced to less than 5 min. Results are therefore provided to the clinician much faster (usually on the day the sample arrives at the clinical laboratory) than with the usual microscopic techniques [7]. Nevertheless, results are only qualitative, and the parasite load cannot be estimated which limits the relevance of the NSP assay for therapeutic follow-up. The invalid results after the first run reached 6.6% (11/156) in this study, which is a slightly higher rate than that observed in the study by Hartuis et al. (2%) [7]. This phenomenon seems to be random and probably related to the presence of PCR inhibitors. In most cases (8/9), a retesting of the sample provided an interpretable result.

In addition, it is difficult to know whether it is still necessary to repeat parasite stool examination when performing the NSP assay as recommended for microscopic methods due to their low overall sensitivity and the intermittent shedding of some parasites [1]. The need to repeat examinations is controversial among populations in which the prevalence of infections is low [20]. According to IDSA guidelines, one option for cost-effective testing may consist of assessing a second and then a third specimen only when the first/second is negative and the patient remains symptomatic [21]. This strategy would limit the cost of diagnosis by NSP assay for a patient. Indeed, the cost of this test, which is higher than that of routine microscopic techniques, may be an obstacle to the implementation of this system in medical laboratories. However, the initial training of technicians and the technical time required are reduced, which may offset the unit cost of the cartridges. In addition, the NSP assay makes it easier to meet laboratory accreditation requirements by reducing inter-operator variability and facilitating skill maintenance.

The results and interpretations of this study are limited by the retrospective design with the pre-selection of samples. Nevertheless, this strategy provided a large number of stools containing protozoans with a wide variety of species, allowing a better assessment of NSP sensitivity.

Given the rarity of some targets, the use of frozen samples was necessary to ensure a sufficient number of positive samples. Comparative analysis between fresh and frozen samples showed no significant difference in the detection sensitivity of the NSP assay. The use of frozen samples does not appear to be the cause of the observed lack of sensitivity of the NSP assay compared to routine qPCR techniques. However, some protozoa remain poorly represented or absent (*Balantioides coli*) from this study, which is inadequate in terms of providing a conclusion about the diagnostic performance of the NSP assay for these targets. Our study was not designed to evaluate the performance of the NSP assay for helminth detection, as the number of positive stool samples available was insufficient to obtain reliable results. However, these performances have already been evaluated by Hartuis et al. for the most frequently observed helminths. Despite a low number of positive samples, the NSP assay showed good sensitivity and specificity for *Schistosoma mansoni*, *Taenia* spp., *Ascaris* sp., *Enterobius vermicularis*, *Strongyloides stercoralis* and hookworms. Nevertheless, weak performance was obtained for the detection of *Trichuris* sp. [7].

## 5. Conclusions

Finally, the overall performance of the NSP assay in the diagnosis of gastrointestinal protozoa seems to be better than or at least equivalent to that observed with microscopy but lower than that obtainable with conventional targeted qPCR. Except for *Trichuris* sp., the study by Hartuis et al. also reported a high detection performance for the most common helminths in comparison with microscopy [7]. This method could therefore represent an interesting alternative for non-specialised laboratories wishing to switch from a microscopy-based technique to an easy-to-use and easy-to-implement molecular method. However, for certain specific targets or in the case of diagnostic failure despite a strong clinical suspicion, a request for expertise in a specialised centre (targeted PCR, specific technique) could prove useful especially for the parasites less well-detected by the NSP assay. Some rare targets have yet to be evaluated on a larger scale.

## Figures and Tables

**Table 1:** Performance of the Novodiag^®^ Stool Parasites Assay (NSP) Compared to Microscopy Techniques (Micro) (*n* = 156).

Targets	Number of Samples with				Se (%)	Sp (%)	Concordance (%)	Coef. Kappa
	Micro (+) NSP (+)	Micro (−) NSP (−)	Micro (+) NSP (−)	Micro (−) NSP (+)				
*Giardia intestinalis*	23	126	4	3	85.2%	97.7%	95.5%	0.84
*Blastocystis hominis*	29	98	1	28	96.7%	77.8%	81.4%	0.55
*Entamoeba histolytica ^a^*	3	147	0	6	100%	96.1%	96.1%	0.48
*Dientamoeba fragilis*	1	132	1	22	50%	85.7%	85.3%	0.06
*Cyclospora cayetanensis*	2	154	0	0	100%	100%	100%	1
*Cystoisospora belli*	0	155	1	0	0%	100%	99.4%	NC

*^a^—*E. histolytica species was confirmed by Amplidiag^®^ Stool Parasites real-time PCR kit. Se—Sensitivity; Sp—Specificity; NC—Cohen’s kappa not calculable (zero values).

**Table 2:** Performance of the Novodiag^®^ Stool Parasites (NSP) Assay Compared to Routine Molecular Biology Techniques (PCR) (*n* = 164).

Targets	Number of Samples with				Se (%)	Sp (%)	Concordance (%)	Coef. Kappa
	PCR (+) NSP (+)	PCR (−) NSP (−)	PCR (+) NSP (−)	PCR (−) NSP (+)				
*Giardia intestinalis ^a^*	26	127	11	0	70.3%	100%	93.3%	0.78
*Dientamoeba fragilis ^a^*	24	126	14	0	63.1%	100%	92.1%	0.74
*Entamoeba histolytica ^a^*	9	151	4	0	69.2%	100%	97.6%	0.80
*Cryptosporidium* spp. *^a^*	12	144	8	0	60.0%	100%	95.1%	0.72
*C. parvum*	10	152	2	0	83.3%	100%	98.8%	0.90
*C. felis*	1	162	1	0	50.0%	100%	99.4%	0.67
*C. canis* ^b^	0	163	1	0	0%	100%	99.4%	NC
*C. hominis*	1	161	2	0	33.3%	100%	98.8%	0.50
*Enterocytozoon bieneusi* ^c^	9	155	0	0	100%	100%	100%	1
*Encephalitozoon* sp. ^c^	0	159	5	0	0%	100%	97.0%	NC

*^a^—*Detection by Amplidiag^®^ Stool Parasites real-time PCR kit. ^b^*—*Species not targeted by NSP assay. ^c^*—*Detection by in-house microsporidia PCR [13]. Se—Sensitivity; Sp—Specificity; NC—Cohen’s kappa not calculable (zero values).

**Table 3:** Sensitivity of Microscopy and the Novodiag^®^ Stool Parasites Assay (NSP) using Amplidiag^®^ Stool Parasites Real-Time PCR Assay as Reference Technique (*n* = 156).

	Sensitivity (%) (Reference = PCR) (*n* = 156)		
	By Microscopy	By NSP	*p*-Value
*Giardia intestinalis*	27/37 (72.9%)	26/37 (70.3%)	0.797 *^a^*
*Dientamoeba fragilis*	2/37 (5.4%)	23/37 (62.9%)	<0.001 *^a^*
*Entamoeba histolytica*	3/13 (23.1%)	9/13 (69.2%)	0.018 *^a^*

*^a^*—chi-square test.

## Data Availability

Not applicable.

## Supplementary Materials

The following supporting information can be downloaded at: https://www.mdpi.com/article/10.3390/pathogens12070889/s1, Table S1: Targets of the Novodiag^®^ Stool Parasites (NSP) assay.
