# Supplementary material for: Evaluation of the Performance of the Novodiag® Stool Parasites Assay for the Detection of Intestinal Protozoa and Microsporidia
Source: Pathogens. 2023 Jun 29;12(7):889. doi: 10.3390/pathogens12070889 (PMC10385646; doi:10.3390/pathogens12070889)
Supplement: Supplementary file 1 [file pathogens-12-00889-s001.zip › pathogens-2458995-supplementary.pdf]

Supplementary Data

Table S1. Targets of the Novodiag® Stool Parasites (NSP) assay.

Protozoa targeted by the NSP
Balantidium coli
Blastocystis spp.
Cryptosporidium spp.[a]
Cyclospora cayetanensis
Cystoisospora belli
Dientamoeba fragilis
Entamoeba histolytica
Giardia intestinalis
**Microsporidia targeted by the NSP**
Encephalitozoon spp.[b]
Enterocytozoon bieneusi
**Helminths targeted by the NSP**
Ancylostoma duodenale
Ascaris lumbricoides / suum
Clonorchis sinensis / Opisthorchis spp. / *Metorchis* spp.
Diphyllobothrium latum / nihonkaiense
Enterobius vermicularis
Fasciola spp.[c]
Fasciolopsis buski
Hymenolepis diminuta
Hymenolepis nana
Necator americanus
Schistosoma mansoni
Schistosoma spp.[d]
Strongyloides stercoralis
Taenia saginata / asiatica
Taenia solium
Trichuris spp.

[a] — Detection of at least C. hominis, C. meleagridis, C. parvum, C. ubiquitum and Cryptosporidium spp. Chipmunk genotype I. [b] — Detection of at least E. cuniculi, E. hellem and E. intestinalis. [c] — Detection of F. hepatica and F. gigantica. [d] — Detection of at least S. bovis, S. curassoni, S. edwardiense, S. haematobium, S. hippopotami, S. incognitum, S. intercalatum, S. japonicum, S. leiperi, S. malayensis, S. mansoni, S. margrebowiei, S. mattheei, S. mekongi and S. sinensium.
